# Discovery and Characterization of a Metastable Cubic Interstitial Nickel–Carbon System with an Expanded Lattice

**DOI:** 10.1021/acsnano.4c15300

**Published:** 2025-01-06

**Authors:** Albert Gili, Martin Kunz, Daniel Gaissmaier, Christoph Jung, Timo Jacob, Thomas Lunkenbein, Walid Hetaba, Kassiogé Dembélé, Sören Selve, Reinhard Schomäcker, Aleksander Gurlo, Maged F. Bekheet

**Affiliations:** † Faculty III Process Sciences, Institute of Materials Science and Technology, Chair of Advanced Ceramic Materials, Technische Universität Berlin, Straße des 17. Juni 135, 10623 Berlin, Germany; ‡ Institut für Chemie, Technische Universität Berlin, Sekretariat TC 8, Straße des 17. Juni 124, 10623 Berlin, Germany; § Helmholtz-Zentrum Berlin für Materialien und Energie, 14109 Berlin, Germany; ∥ Advanced Light Source, Lawrence Berkeley National Laboratory, Berkeley, California 94720, United States; ⊥ Institute of Electrochemistry, Ulm University, Albert-Einstein-Allee 47, 89081 Ulm, Germany; # Helmholtz-Institute Ulm (HIU) Electrochemical Energy Storage, Helmholtzstr. 11, 89081 Ulm, Germany; ∇ Karlsruhe Institute of Technology (KIT), P.O. Box 3640, 76021 Karlsruhe, Germany; ○ Department of Inorganic Chemistry, Fritz-Haber-Institut der Max-Planck-Gesellschaft, 14195 Berlin, Germany; ◆ Abteilung Heterogene Reaktionen, Max-Planck-Institut für chemische Energiekonversion, Stiftstr. 34-36, 45470 Mülheim an der Ruhr, Germany; ¶ Center for Electron Microscopy (ZELMI), Technische Universität Berlin, Straße des 17. Juni 135, 10623 Berlin, Germany

**Keywords:** solid solution, carbide, in situ XRD, TEM, nanoparticle, catalysis

## Abstract

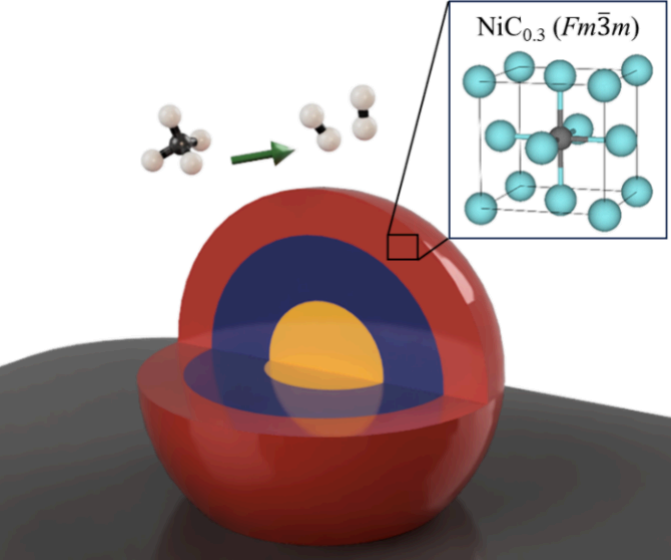

Metastable, *i.e.*, kinetically favored but thermodynamically not stable, interstitial solid solutions of carbon in iron are well-understood. Carbon can occupy the interstitial atoms of the host metal, altering its properties. Alloying of the host metal results in the stabilization of the FeC_*x*_ phases, widening its application. Pure nickel finds niche applications, mainly focusing on catalysis, while nickel alloys are widely applied, *e.g.*, in gas turbines, reactors, and seawater piping. Nickel carbide (Ni_3_C) is the well-known stable Ni–C system displaying a rhombohedral (*R*3̅*c*) crystal structure. Some reports describe an elusive cubic Ni–C system, observed during certain catalytic reactions occurring on nickel and formed by the occupation of the interstitials of the metal with carbon: to date, the stabilization and characterization of this phase have not been accomplished. Hereby, we report on the synthesis of a cubic metastable NiC_*x*_ phase using chemical vapor deposition of methane on supported nickel nanoparticles. The structure was predicted by DFT/ReaxFF, synthesized and monitored with *in situ* time-resolved synchrotron XRD, and experimentally confirmed by Rietveld refinement and (S)TEM-EELS under ambient conditions. The results show an *Fm*3̅*m* phase with a lattice parameter of *a* = 3.749 ± 0.037 Å at room temperature, with the highest ever reported atomic percentage of carbon occupying the octahedral interstices of 23.1%, resulting in a NiC_0.3_ phase. The degree of occupation of the interstitial voids by carbon can be controlled, enabling the tuning of the host metal’s *d*-spacing and composition, highlighting the applicability of this synthesis route for catalytic nanoparticle preparation.

## Introduction

Iron and carbon constitute the most studied example of an interstitial solid solution: carbon can occupy the interstitials of the host iron structure, distorting its lattice.^1^ Quenching leads to the (meta)stabilization of different phases like martensite (*P*4/*mmm*), bainite (*P*4/*mmm* or *Pm*3̅*m*), or pearlite (*Pnma*), which display distinct properties compared to simple iron. These processes have been applied for thousands of years: today, steel is one of the most used materials globally. In comparison to iron, the story of nickel and carbon is less ubiquitous.

Metallic nickel possesses a cubic structure (*Fm*3̅*m*), whereas the known nickel carbide (Ni_3_C) exhibits a trigonal (*R*3̅*c*) structure with an additional ordered sublattice of interstitial carbon.^2^ Several reports in the literature describe the formation of a solid solution of carbon in the cubic nickel during specific chemical processes like the chemical vapor deposition (CVD) of hydrocarbons to form carbon nanotubes/nanofibers (CNT/CNF)^3−5^ or during the dry reforming of methane (DRM).^6,7^ During CVD, CH_4_ (or other C-containing precursors) decomposes to produce H_2_ and surface carbon atoms. These can occupy the interstitial sites of the metal host, forming a solid solution and expanding the crystal’s unit cell.^4,7^ Graphitic CNT/CNF formation follows the exsolution of the dissolved carbon under certain conditions.^3^ Understanding the carbon diffusion mechanisms and the NiC_*x*_ intermediates is of key importance for rational catalyst design.^7,8^ Indeed, theoretical work already foresaw the existence of a metastable cubic Ni–C system in the transformation sequence from *fcc* Ni to *hcp* Ni_3_C, predicting that carbon atomic contents above 25% in the *fcc* Ni_4_C would lead to instability and yield the *hcp* Ni_3_C.^8^ Experimentally, the work successfully used Au as a substrate for the growth of Ni and later carbonization to stabilize the metastable NiC_*x*_ compounds due to the lattice match with the Au support.

The increasing price of noble metals combined with the unique capability of nickel to enable new catalytic pathways has stimulated interest in this transition metal for organic chemistry,^9^ heterogeneous,^10^ electro-,^11^ and photocatalysis.^12^ Using substitutional and intermetallic alloys enables engineering the electronic configuration of multimetallic catalytic nanoparticles, modifying the adsorption and reaction energies of specific catalytic pathways.^10,13,14^ Besides, the inclusion of heteroatoms in the interstices of catalytic nanoparticles strongly affects the subsurface chemistry and modifies the product selectivity.^15−19^ An example is hydrogenation reactions: if present, C occupies the interstitials of Pd (or Ni_3_Zn^16^) during alkyne semihydrogenation, minimizing the population of hydrogen in the subsurface and avoiding overhydrogenation of the reaction intermediates.^20^

In this work, we report the prediction, synthesis, and characterization of supported cubic NiC_*x*_ nanoparticles synthesized by CVD of CH_4_. DFT/ReaxFF calculations predict the structure and point out the preferred occupation of octahedral interstitials. Synthesis of the NiC_*x*_ structures was followed *in situ* using synchrotron X-ray diffraction (XRD). Stabilizing the interstitial alloy and anchoring carbon in the nickel nanoparticles allows further investigation of the structures at room temperature. Diffraction combined with transmission electron microscopy (TEM) and scanning transmission electron microscopy-electron energy loss spectroscopy (STEM-EELS) demonstrates the metastable presence of a cubic supersaturated NiC_0.3_ phase at room temperature. The discovery of this phase encourages further investigation to be applied as a synthesis route to engineer the electronic configuration, tune the *d*-spacing, and control the subsurface chemistry of supported nickel nanoparticles for catalysis.

## Results and Discussion

**Figure 1:**
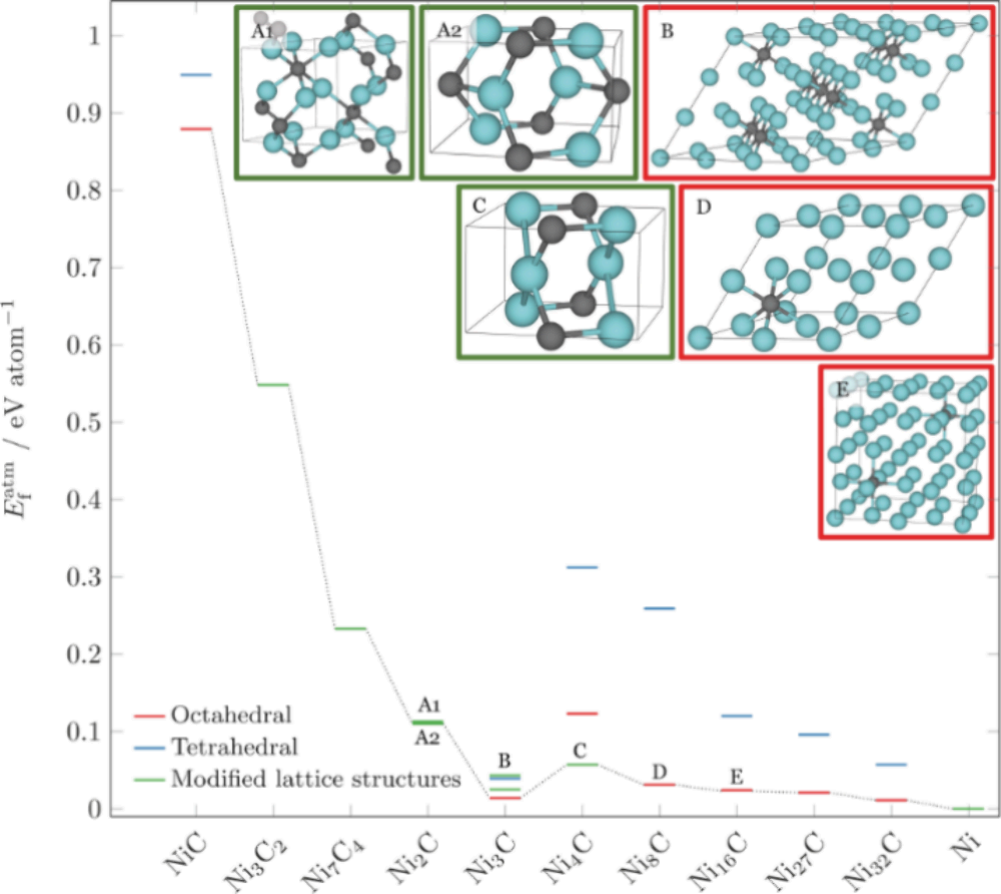
Formation energies per atom (*E*_f_^atm^) obtained with DFT/ReaxFF for several NiC_*x*_ in tetrahedral (blue) and octahedral (red) interstitials as well as selected modified lattice structures^22^ (green) at 800 °C.

Figure 1 shows the obtained (Helmholtz free) energies of formation per atom for various NiC_*x*_ structures at 800 °C (extended data are shown in Tables S1 and S2 and Figures S1–S3). The results show a preferential occupation of the octahedral interstitial sites (as previously predicted^21^) by C and an energetically preferred cubic Ni_3_C. For a small number of doped carbon atoms (<Ni_3_C), it is energetically favorable to form octahedral Ni_*x*_C structures as well. For structures with carbon content larger than that of Ni_3_C, the space groups of the modified lattices become more stable. For the modified lattice structures of Ni_2_C A1 and A2, an energetically degenerate state occurs. Our results are an extension of the possible structures previously reported^8^ and agree with the relative stability of Ni_3_C and predict its existence.

**Figure 2:**
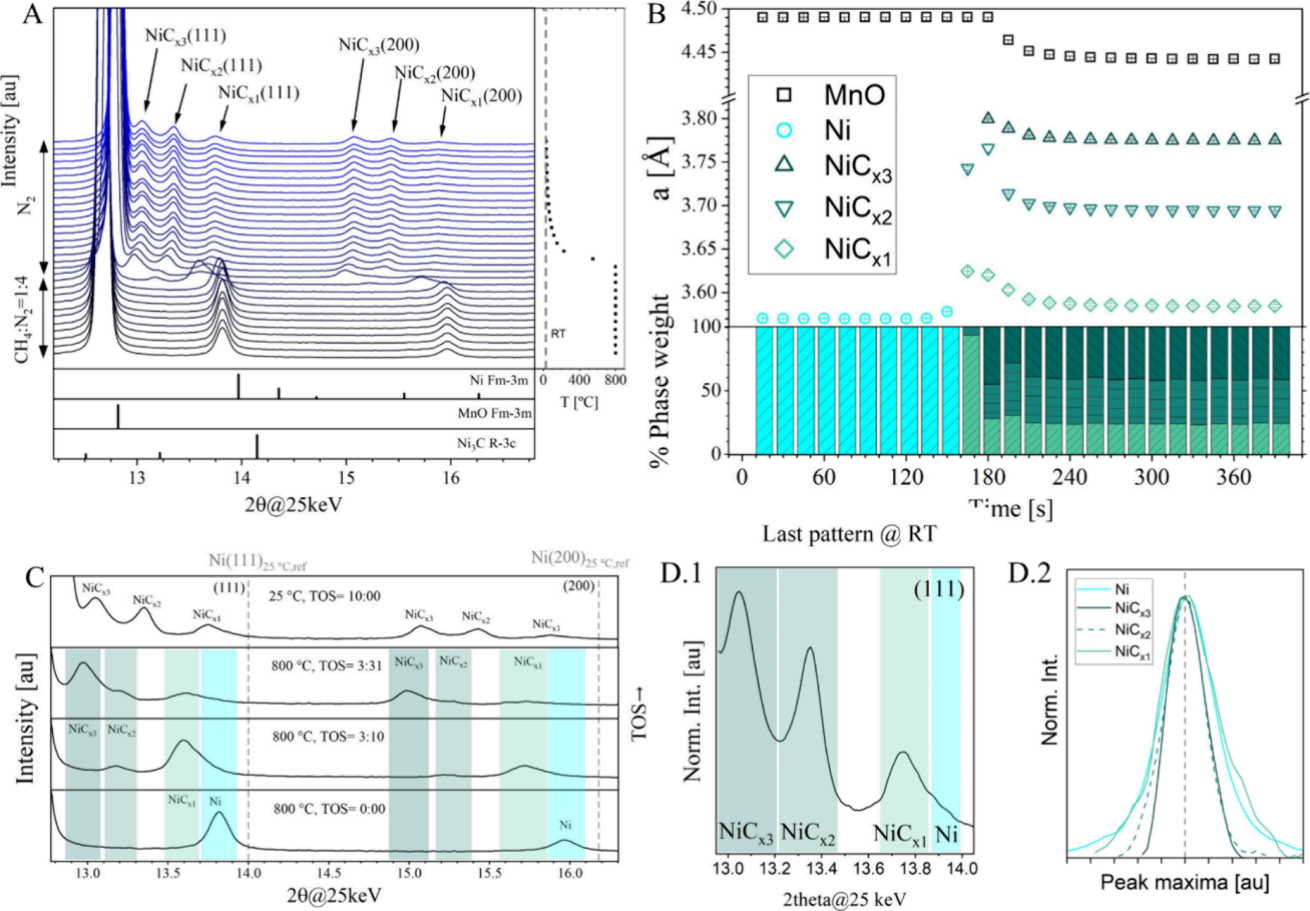
Phase evolution of the Ni and NiC_*x*_ phases from *in situ* XRD during the CVD of CH_4_. Panel (A) shows the evolution of the Ni/NiC_*x*_(111) and (200) reflections: the stacked patterns evolve from black to blue with TOS. Reference graphs belong to Ni (*Fm*3̅*m*, 00-004-0850), MnO (*Fm*3̅*m*, 01-088-0424), and Ni_3_C (*R*3̅*c*, 01-072-1467). Panel (B) shows the lattice parameter and weight fraction of the Ni-containing phases (relative to the Ni-containing phases exclusively) as a function of the TOS obtained by Rietveld refinement. Panel (C) displays a magnification of the Ni(111) and (200) zones of selected patterns. Panel (D.1) shows the individually normalized intensity of the NiC_*x*1–3_ (111) reflection of the last pattern at RT. Panel (D.2) shows the comparison of peak shape of the (111) reflections of Ni (800 °C) and the NiC_*x*_ (last pattern, RT, the same as D.1). The reflections’ maxima are displaced and the intensity normalized to allow for shape comparison.

The synthesis of the predicted cubic nanoparticles of NiC_*x*_/MnO was performed and monitored in situ using XRD (the reasons for using MnO as a support are clarified in the Methods section). Figure 2A shows the patterns obtained during CVD at 800 °C and posterior quenching (see Figure S4 for the initial structure after the reduction step/proof of the absence of NiO and Figure S5 for the full experiment): the patterns are stacked and progressively turn from black to blue (bottom up) with time on stream (TOS). Decomposition of CH_4_ on the Ni nanoparticles at 800 °C results in the intercalation of C atoms in the octahedral interstices of the metal (in contrast to tetrahedral occupation as theoretically proven in Figure 1), expanding its unit cell as evidenced by the shift toward lower 2θ of the nickel reflections.^7^ Note that this expansion cannot be caused by the endothermic CH_4_ decomposition reaction. The formation of NiC_*x*_'s is due to the difference in energy barrier between CH_4_ decomposition,^23^ a reaction occurring on the external surface of the particles, and C diffusion toward the core of the nanoparticles, resulting in an onion-type multiphase structure with increasing C content from core to shell.^7^ Due to the on-the-fly analysis during data acquisition, the oven was quenched (−6.5 °C·s^–1^, see the Methods section) as soon as the expanded reflections were observed. The rapid drop in temperature shifts the reflections to higher angles due to thermal contraction, and even after a few minutes at room temperature, the NiC_*x*_ phases remain detectable. The lack of the main graphite reflection (002) (*P*6_3_/*mmc*, PDF No. 00-041-1487) at 2θ = 8.42 (25 keV) (see Figure S5) at the end of the experiment demonstrates the absence of significant graphitic structures, suggesting the success of the quenching procedure. This procedure hinders kinetically the otherwise thermodynamically favored exsolution of C-structures upon cooling. It is worth noting that our previous works showed that the formation of graphitic carbon during the CVD process is always accompanied by the exsolution of carbon from the NiC_*x*_ phases.^6,7^ During the whole process, nickel and the NiC_*x*_ phases maintain their cubic (*Fm*3̅*m*) structure: the *R*3̅*c* structure belonging to Ni_3_C remains undetectable. The lattice parameter obtained by Rietveld refinement of the *in situ* data (Figure 2A) *vs* TOS is shown in Figure 2B: at room temperature, NiC_*x*3_ displays a value of 3.7753(7) Å, NiC_*x*2_ displays a value of 3.6943(7) Å, and NiC_*x*1_ displays a value of 3.5844(15) Å. An estimation of the carbon content can be obtained by combining experimental values of lattice parameters and the ReaxFF simulations, as previously done.^7^ Thus, phase compositions of NiC_0.30_ (NiC_*x*3_), NiC_0.24_ (NiC_*x*2_), and NiC_0.05_ (NiC_*x*1_) are obtained. The carbon content of all these compounds is above the experimental limit of solubility in bulk nickel^23^; hence the term supersaturated. It is worth noting that the estimated lattice parameter of the cubic NiC_*x*_ phase reported by Kang et al.^8^ was 3.637(5), lower than those of the NiC_*x*3_ and NiC_*x*2_ reported in this work. Therefore, the maximum solubility of C in the *fcc* lattice of Ni is experimentally observed in the structures reported here. The relative weight percentage of each Ni-containing phase obtained from refinement is shown at the bottom of Figure 2B, highlighting the total disappearance of metallic nickel and the major fraction of NiC_*x*3_ (the most external NiC_*x*_). The symmetry of the NiC_*x*_ reflections changes compared to that of metallic Ni. The shift of the peak maxima’s position is related to the expansion of the unit cell caused by the intercalation of C: lower 2Θ angle means more expanded cell, *i.e.*, more C in the NiC_*x*_ structure. Figure 2D.2 shows the Ni (at 800 °C before the CVD process) and the NiC_*x*_ (last pattern@RT) normalized (111) reflections (in both intensity and peak maxima position). The NiC_*x*1_ reflection exhibits a very similar peak shape compared to that of Ni, with very similar Gaussian and Lorentzian integral breadths but with the appearance of an asymmetric shoulder right of the maxima: this is most probably created by the existence of a solid-solution gradient to lower C-contents or even by the presence of “unreacted” metallic Ni. Note that the presence of a solid-solution gradient results in a Gaussian-type of broadening, similar to that of microstrain.^24^ A similar shoulder is observed left of the maxima of the NiC_*x*2_ reflection, which could be caused again by the presence of a solid-solution gradient. A decrease of the Lorentzian integral breadth in the NiC_*x*2_ and NiC_*x*3_ compared to the initial Ni is most probably caused by crystallite size growth: the presence of defects like dislocations, twin faults, stacking faults, and layer mistakes would induce Lorentzian broadening to the peak shape, which is not observed in our experiment. The sample was recovered for characterization with TEM.

**Figure 3:**
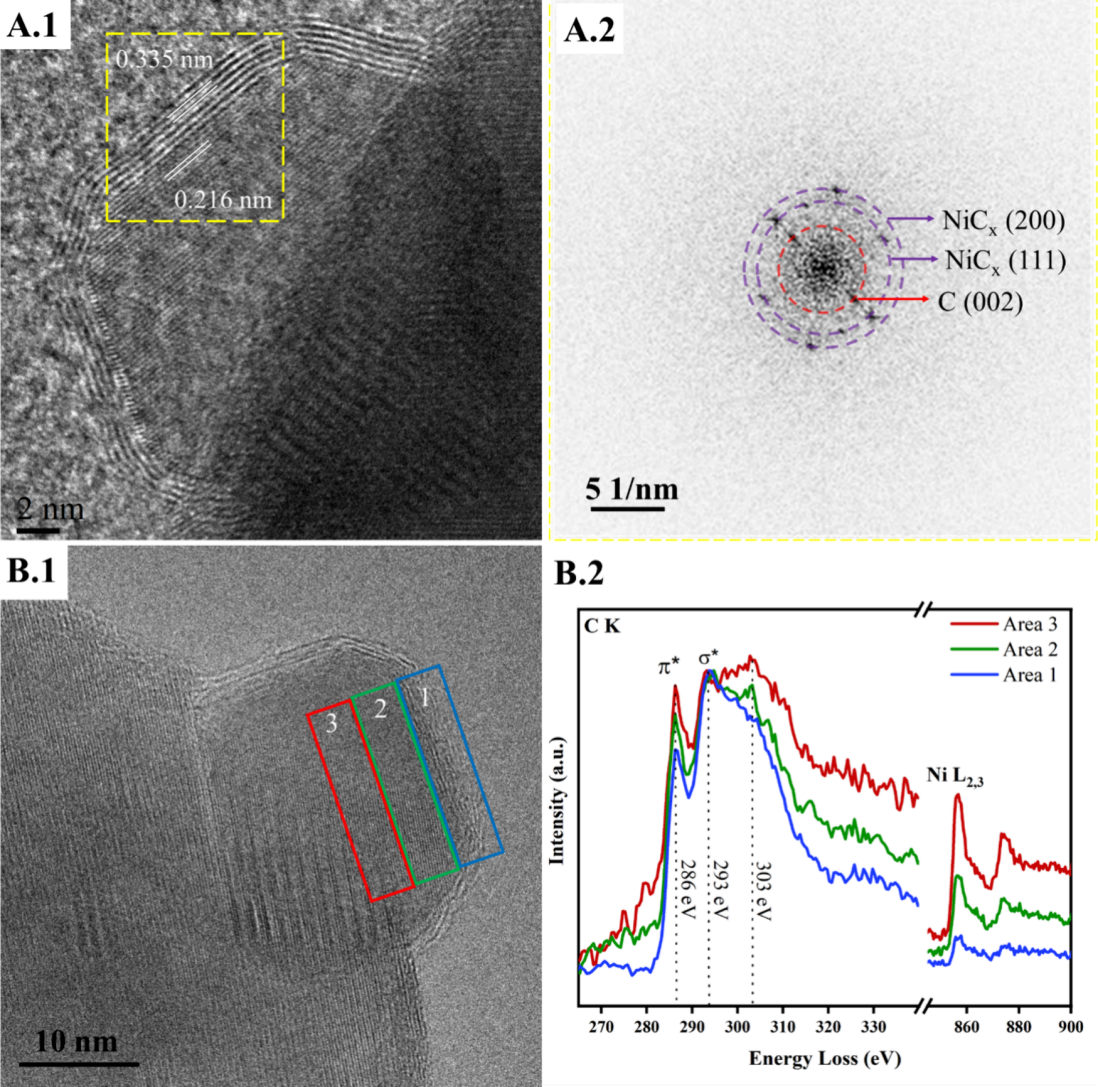
Structural and chemical characterizations with TEM-STEM/EELS. (A.1) HR-TEM image of a NiC_*x*_ sample with an interplanar distance obtained from FFT. (A.2) FFT on the inset in (A.1), with interplanar distances belonging to C(002) = 0.335 nm, NiC_*x*_(111) = 0.216 nm, and NiC_*x*_(200) = 0.185 nm. (B.1) TEM image showing a nanoparticle with a carbon encapsulating layer and 3 zones of analysis with EELS. (B.2) EELS spectra of image B.1, showing the nickel and carbon edge of the three areas depicted in (B.1). The EEL spectra were Fourier ratio deconvolved to remove the effects of plural scattering.

Figure 3 shows the morphological and chemical characterization of the samples performed using TEM and EELS (other nanoparticles and scales shown in Figure S6). The findings highlight the absence of CNTs/CNFs (Figure S6A). Most of the nickel nanoparticles display a thin graphitic structure (Figure 3A.1), as indicated by its interplanar distance *d*_(002)_ of ∼0.335 nm, or an amorphous carbon (Figure S6B1,B2) layer covering their outer surface, most probably generated upon minor carbon exsolution during quenching.^5,25^ A few graphitic structures can be observed, as evidenced by selected area electron diffraction (SAED, Figure S7). An image of NiC_*x*_/MnO is shown in Figure 3A.1. The corresponding fast Fourier transform (FFT, Figure 3A.2) shows an interplanar distance of 0.216 nm, characteristic of the (111) plane of the supported NiC_0.3_ or NiC_0.24_ phases (see Table S4). This *d*-spacing is 6.2% above the reported value for metallic nickel (PDF No. 00–004–0850), highlighting the expanded cell. Calculation of the lattice parameter of this value leads to *a* = 3.741 Å, in good agreement with values obtained from refinement of the XRD data. STEM-EELS analysis of a typical NiC_*x*_ (Figure 3B2) particle allows determining qualitative changes in the carbon content and the structure of nickel along the radial direction of the particle (colored areas in Figure 3B.1). There is an increase of the C/Ni ratio in the radial direction from core to shell, most probably caused by the encapsulation of the particle by graphitic carbon and/or the increase of the C content in the NiC_*x*_ phase. The σ*/π* ratio of the C signal increases in the radial direction from core to shell, which could be related to an increase in the C concentration in the NiC_*x*_ phase. However, its interpretation is not straightforward as carbon is known for its anisotropicity and the ratio can change if the carbon structure is oriented differently to the electron beam. A peak at 303 eV is observed in areas 3 and 2, which decreases from core to shell. A similar peak has been previously reported at energy losses of 302 and 305 eV and has been attributed to carbide formation in the Al_2_MgC_2_ system.^26^ An additional proof of the formation of a solid solution of C in Ni evolves from the comparison of the Ni L_2,3_-edge acquired on a NiC_*x*_ sample and a Ni-metal reference sample (Figure S8). The spectrum of the NiC_*x*_ sample is shifted 1 eV toward higher energy losses compared to the spectrum of the Ni-metal. The same shift was previously observed in Ni 2p XPS^5^ and in the Fe L_2,3_ electron energy-loss edge of iron carbide.^27^

The NiC_*x*_ structures are metastable: Figure S9 shows a nanoparticle from another experiment performed with the same material and procedure but quenched before the maximal expansion was achieved. The original particle (Figure S9A.1,B) shows the absence/a very thin carbon external shell, and *d*-spacings ∼5% expanded compared to the reference Ni. During observation in the microscope, the particle shrunk (from a diameter of 33.33 to 28.63 nm, relative shrinkage of 14.1%), and an external shell of approximately 4.79 nm amorphous C was formed. This phenomenon was caused by the electron beam, which resulted in the exsolution of the C dissolved from the interstitials of the Ni nanoparticle.

Previous observations of similar *fcc* NiC_*x*_ phases were reported,^5^ but recovery and characterization are done for the first time in this work. We envision the methodology described here as a tool to produce supported metal nanoparticles with an engineered electronic configuration, lattice parameter, and tailored subsurface chemistry. The application of such systems (including nickel and some nickel-containing alloys as starting supported nanoparticles) has great potential for heterogeneous, electro-, and photocatalysis. Indeed, some notorious cases have already been reported.^16^ The catalyst support can be modified to adapt to specific catalytic systems, while several methods can be applied to impregnate controlled-sized metal nanoparticles. The size of the initial particles strongly affects the carbon solubility limit^28^ and, as a consequence, the final composition, lattice parameter, and electronic configuration. Through precise control of the CVD conditions applied, namely, carbon-precursor type, flow and partial pressure, temperature,^7^ and exposure time, specific structures can be obtained.

## Conclusions

We report on the prediction of a NiC_*x*_*Fm*3̅*m* structure using DFT/ReaxFF, its synthesis in supported nanoparticulate form using time-resolved synchrotron-based *in situ* XRD, and the later *ex situ* characterization using electron microscopy. Theoretical and experimental results show a *fcc* Ni crystal structure with C atoms occupying the octahedral interstitials to yield metastable NiC_0.3_. The thorough characterization of this phase is done for the first time; we envision the suggested methodology as a pathway to create supported metal nanoparticles with tailored composition, lattice parameter, and subsurface chemistry.

## Methods

### 5% Ni/MnO Synthesis

A procedure previously reported in ref (6) was followed. Briefly, the NiO/MnO_*x*_ catalyst precursor was synthesized using the coprecipitation method. An aqueous solution containing the proper amounts of nickel acetate tetrahydrate (Ni(OCOCH_3_)_2_·2H_2_O) and manganese nitrate tetrahydrate (Mn(NO_3_)_2_·4H_2_O) was mixed under stirring with an aqueous solution of NaHCO_3_ and NaOH with a pH ≥ 10 to induce precipitation. The resulting solid was dried at 80 °C and subsequently calcined for 4 h at 750 °C. To achieve the catalyst during the in situ XRD analysis, a flow of pure H_2_ (GHSV = 54,000 L h^–1^ kg_cat_^–1^) was applied at 500 °C. The reduction of the MnO_*x*_ support results in the exsolution of metallic nickel nanoparticles which decorate the surface of the MnO support. The reasons for the use of MnO as a support are (i) to produce exsoluted Ni nanoparticles with controlled size and (ii) due to the previous application of the catalyst to the dry reforming of methane,^6,7^ studies which initiated the present work. MnO is inert for CH_4_ decomposition.

### Characterization

In situ XRD experiments were performed on beamline 12.2.2 of the Advanced Light Source (ALS) synchrotron facility in Berkeley, CA, USA. The setup and technique details have been previously described in refs (29−31). Shortly, a set of mass flow controllers allow controlling the flow rate and composition of the gas stream. Around 1 mg of the powder sample is contained inside a quartz capillary and assembled into the setup. Gas delivery occurs by using a tungsten carbide filament. The reaction temperature is controlled with an S-type thermocouple and using two IR lamps connected to a PID controller *via* Labview software. A 2D PerkinElmer detector allows for acquiring high-quality patterns using short acquisition times. A typical experiment consists of (i) heating steps (N_2_ flow, 1 °C·s^–1^), (ii) the reduction step to reduce the NiO/Mn_2_O_3_ solid solution to produce Ni/MnO (1 N mL·min^–1^ flow of H_2_, see Figure S4 for structural information: Ni is in metallic form at the beginning of the experiment, no NiO could be detected), the CH_4_ decomposition (800 °C, reactant mixture of CH_4_:N_2_ = 1:4 and GHSV of 60,000 L h^–1^ kg_cat_^–1^, time 0:00 belongs to the injection of CH_4_ in the gas feed) using 5% Ni/MnO as a material precursor to yield the NiC_*x*_/MnO compound, and (iv) the final quenching. In order to quench the sample and immobilize the carbon content, short acquisition times of 20 s pattern^–1^ were used to observe changes in the position of the nickel (111) and (200) reflections while analyzing the data “on-the-fly” using Dioptas software. When the reflection shift was observed, the oven power supply was interrupted (set to 0), and the reactant flow rate was stopped while continuously acquiring patterns. Applying this methodology and for the experiments reported, the cooling rate was approximately −6.5 °C s^–1^ between 800 and 100 °C. The cooling rate was nonlinear due to the difficulty in removing heat from the setup at lower temperatures due to the decreased temperature gradient between the SiC tube and the air. At room temperature, pure N_2_ was flushed through the sample, and the sample was recovered in an inert gas for later microscopy characterization. Rietveld refinement of the XRD data was performed using FullProf Suite software^32^ and fitting using a Thompson–Cox–Hastings pseudo-Voigt function^33^ with an axial divergence asymmetry function^34^ (Function 7). The sample-to-detector distance for integration and the instrument resolution function for refining were obtained by using a LaB_6_ NIST 660c standard.

*Ex situ* TEM was performed by using two different microscopes. First, a JEOL microscope (JEM-ARM200 CF) operating at 200 kV and equipped with a cold field emission gun (CFEG) and a double Cs corrector for TEM and STEM imaging modes was used. STEM images were acquired using a Gatan high-angle annular dark-field (HAADF) and bright field (BF) detectors, with a probe size of 1.5 nm and a current of 1 nA. STEM-EELS data were acquired using a Gatan imaging filter (GIF) spectrometer (model quantum) with a beam convergence semiangle of 22.8 mrad, a collection semiangle of 54.5 mrad, and a dispersion energy of 0.5 eV per channel. The current was set between 2 × 10^7^ and 9 × 10^7^ e/s, which was verified by using a Faraday cup holder. Second, an image Cs-corrected HR-TEM (FEI Titan 80–300 Berlin Holography Special) microscope with XFEG operated at 300 kV was used. Under the conditions applied in Figure S9, the calculated electron dose rate was 3.3 × 10^9^ electron·s^–1^, with the overall dosage on the very particle between both images being 1.4 × 10^13^.

### DFT Calculations

First-principles calculations have been performed using the Vienna Ab initio Simulation Package (VASP).^35,36^ The projector augmented wave (PAW) method^37^ in the implementation of Kresse and Joubert^35^ was applied for an accurate and efficient calculation of the system’s electronic structure. Exchange correlation effects were described by the Bayesian error estimation functional with van der Waals (BEEF-vdW)^38^ in the framework of the generalized gradient approximation (GGA). According to the scheme of Monkhorst and Pack, a *k*-point mesh density of 0.15 Å^–1^ was used to sample the Brillouin zone (BZ). For an accurate expansion of the one-particle electron wave functions into a plane-wave basis set, a cutoff energy of 750 eV was chosen. The calculations were considered to be converged once the total energy difference was less than 1 × 10^–7^ eV and the norms of all the forces were smaller than 1 × 10^–4^ eV Å^–1^. After intensive testing, the reactive force field (ReaxFF) of Mueller et al.^39^ was used to determine the entropic contributions. All ReaxFF^40,41^ calculations were performed within the ReaxFF implementation of the Amsterdam Modeling Suite 2019.^42^ The contributions for the respective systems were calculated within the ASE Phonon module framework.^43,44^ Each cell was repeated three times in all spatial directions to minimize the influence of the periodic images, and a displacement value of 0.01 Å for the small displacement method was applied.

The formation energies per formula unit (f.u.) and atom (atm) were calculated by
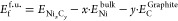
1and
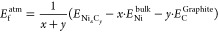
2where *E*_Ni_*x*_C_*y*__ is the total energy of the Ni–C bulk system, *E*_Ni_^bulk^ is the total energy per atom of *fcc*-Ni, and *E*_C_^Graphite^ is the energy of a single C atom in graphite. Internal energy components *U* at *T* = 1073 K have been calculated from the sum of the system’s potential energy *E*_pot_, zero-point energy *E*_ZPE_, and phonon energy contribution *E*_Phonon_ using the following equation:
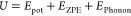
3The Helmholtz free energy at *T* = 1073 K was then determined by
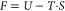
4where *S* is the entropy contribution.

All studied structures are provided as POSCAR files in the Supporting Information.

## Supporting Information Available

Summary of the calculated formation energies; schematic illustration of the studied octahedral Ni–C bulk cells; schematic illustration of the studied tetrahedral nickel NiC_*x*_ bulk cells; schematic illustration of the studied modified nickel NiC_*x*_ lattice structures; summary of the calculated zero-point energy values, energy contribution of the phonons, internal energy values, entropy contributions, and formation energy values; *in situ* XRD patterns of the reduction step; *in situ* XRD patterns of the full CVD experiment; TEM images; SAED analysis; EELS comparison of the Ni L_2,3_-edge acquired on a NiC_*x*_ particle and a Ni-metal reference sample; Ni, C (graphite), and MnO plane distances with corresponding hkl indexes; and comparison of experimental interplanar distances obtained by TEM imaging and XRD-RR analysis (PDF)

## ABBREVIATIONS

CVD: chemical vapor deposition
CNF: carbon nanofiber
CNT: carbon nanotube
EELS: electron energy loss spectroscopy
TEM: transmission electron microscopy
STEM: scanning transmission electron microscopy
SAED: selected area electron diffraction
