# Supplementary material for: Discovery and Characterization of a Metastable Cubic Interstitial Nickel–Carbon System with an Expanded Lattice
Source: ACS Nano. 2025 Jan 6;19(2):2769–76. doi: 10.1021/acsnano.4c15300 (PMC11760165; doi:10.1021/acsnano.4c15300)
Supplement: Supplementary file 1 — nn4c15300_si_001.pdf [file nn4c15300_si_001.pdf]

Supporting information of

# Discovery and characterization of a metastable cubic interstitial nickel-carbon system with expanded lattice

*Albert Gili[1,2,3,4*], Martin Kunz[4], Daniel Gaissmaier[5,6,7], Christoph Jung[5,6,7], Timo Jacob[5,6,7], Thomas Lunkenbein[8], Walid Hetaba[9], Kassiogé Dembélé[8], Sören Selve[10], Reinhard Schomäcker[2], Aleksander Gurlo[1,†], Maged F. Bekheet[1,†]*

[1] *Technische Universität Berlin, Faculty III Process Sciences, Institute of Materials Science and Technology, Chair of Advanced Ceramic Materials, Straße des 17. Juni 135, 10623 Berlin, Germany*

[2] *Institut für Chemie, Technische Universität Berlin, Sekretariat TC 8, Straße des 17. Juni 124, 10623 Berlin, Germany*

[3] *Helmholtz-Zentrum Berlin für Materialien und Energie, 14109, Berlin, Germany*

[4] *Advanced Light Source, Lawrence Berkeley National Laboratory, Berkeley, California 94720, USA*

[5] *Institute of Electrochemistry, Ulm University, Albert-Einstein-Allee 47, 89081 Ulm, Germany*

[6] *Helmholtz-Institute Ulm (HIU) Electrochemical Energy Storage, Helmholtzstr. 11, 89081 Ulm, Germany*

[7] *Karlsruhe Institute of Technology (KIT), P.O. Box 3640, 76021 Karlsruhe, Germany*

[8] *Fritz-Haber-Institut der Max-Planck-Gesellschaft, Departments of Inorganic Chemistry, 14195 Berlin, Germany*

[9] *Max-Planck-Institut für chemische Energiekonversion, Abteilung Heterogene Reaktionen, Stiftstr. 34-36, 45470 Mülheim an der Ruhr, Germany*

[10] *Center for Electron Microscopy (ZELMI), Technische Universität Berlin, Straße des 17. Juni 135, 10623 Berlin, Germany*

**This file includes:**

Figures S1 to S9

Tables S1 to S4

*Table S1. Summary of the calculated formation energies $\mathbf{E_f}$ per formula unit (f.u.) and atom (atm) for several $NiC_x$ systems at T= 0 K. The calculated modified lattice structures have been adopted from Gibson et al. [1]. All energy values are reported in eV.*

| | | Space group | Ni/C ratio | $E_f^{f.u.}$ | $E_f^{atm}$ |
|---|---|---|---|---|---|
| Octahedral | NiC | $Fm\bar{3}m$ | 0.500 | 2.153 | 1.076 |
| | $Ni_3C$ | $Immm$ | 0.750 | 0.660 | 0.018 |
| | $Ni_4C$ | $Pm\bar{3}m$ | 0.800 | 1.006 | 0.201 |
| | $Ni_8C$ | $Fm\bar{3}m$ | 0.889 | 0.629 | 0.070 |
| | $Ni_{16}C$ | $Im\bar{3}m$ | 0.941 | 0.726 | 0.043 |
| | $Ni_{27}C$ | $Fm\bar{3}m$ | 0.964 | 0.836 | 0.030 |
| | $Ni_{32}C$ | $Pm\bar{3}m$ | 0.970 | 0.713 | 0.022 |
| Tetrahedral | NiC | $F\bar{4}3m$ | 0.500 | 1.855 | 0.927 |
| | $Ni_3C$ | $Imm2$ | 0.750 | 1.626 | 0.045 |
| | $Ni_4C$ | $P\bar{4}3m$ | 0.800 | 1.864 | 0.373 |
| | $Ni_8C$ | $F\bar{4}3m$ | 0.889 | 2.540 | 0.282 |
| | $Ni_{16}C$ | $R\bar{3}m$ | 0.941 | 2.287 | 0.135 |
| | $Ni_{27}C$ | $F\bar{4}3m$ | 0.964 | 2.487 | 0.089 |
| | $Ni_{32}C$ | $P\bar{4}3m$ | 0.970 | 2.342 | 0.071 |
| Modified lattice structure | $Ni_3C_2$ | $Pm$ | 0.600 | 2.857 | 0.571 |
| | $Ni_7C_4$ | $Pm$ | 0.636 | 3.825 | 0.348 |
| | $Ni_2C$ ($Pbcn$) | $Pbcn$ | 0.667 | 0.555 | 0.185 |
| | $Ni_2C$ ($Pnnm$) | $Pnnm$ | 0.667 | 0.559 | 0.186 |
| | $Ni_3C$ (bainite) | $P6_322$ | 0.750 | 0.275 | 0.069 |
| | $Ni_3C$ (cemenite) | $Pnma$ | 0.750 | 0.388 | 0.097 |

| | | | | |
|---|---|---|---|---|
| $Ni_4C$ | $P2/m$ | 0.800 | 0.639 | 0.128 |

## Octahedral structures

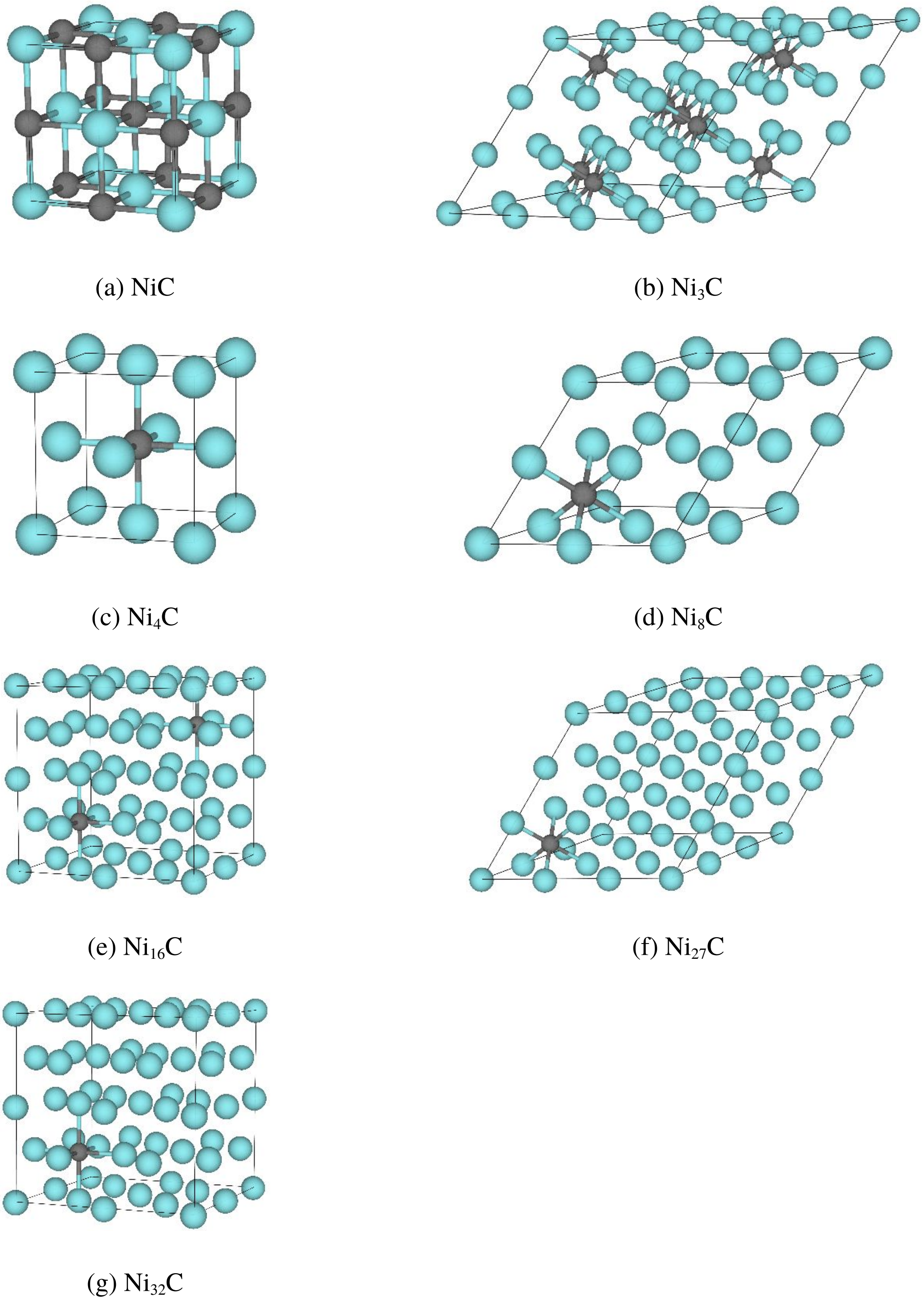


*Figure S1. Schematic illustration of the studied octahedral Ni-C bulk cells. Carbon atoms are shown in grey, and nickel atoms are depicted in cyan.*

## Tetrahedral structures

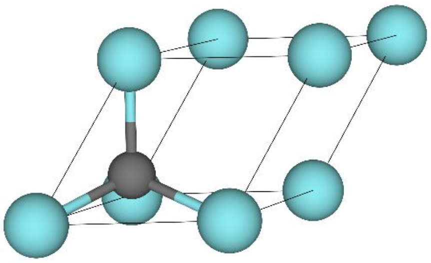

(a) NiC

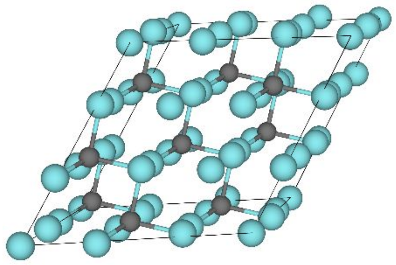

(b) $Ni_3C$

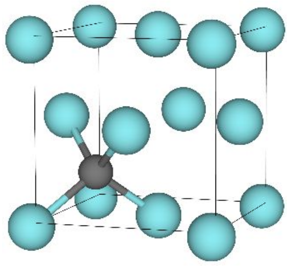

(c) $Ni_4C$

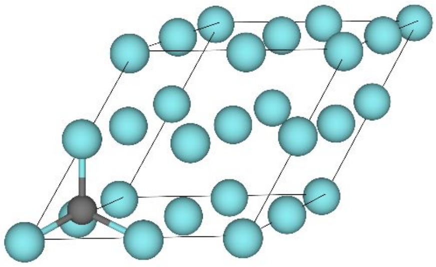

(d) $Ni_8C$

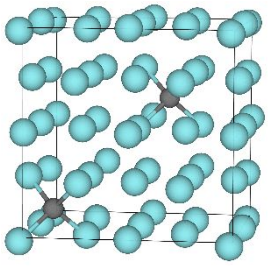

(e) $Ni_{16}C$

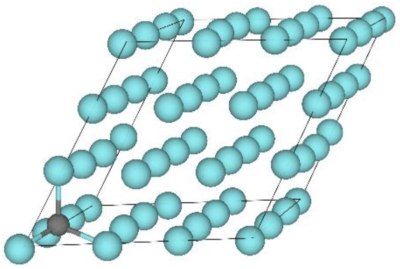

(f) $Ni_{27}C$

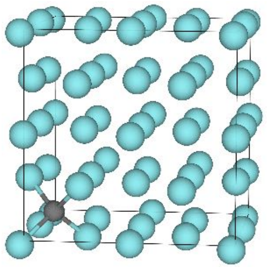

(g) $Ni_{32}C$

*Figure S2. Schematic illustration of the studied tetrahedral nickel $NiC_x$ bulk cells. Carbon atoms are shown in grey, and nickel atoms are depicted in cyan.*

Modified lattice structures [1]

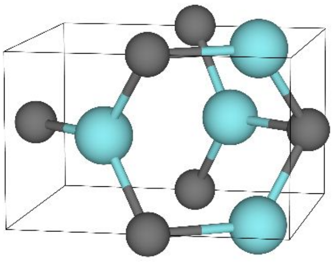

(a) $Ni_3C_2$

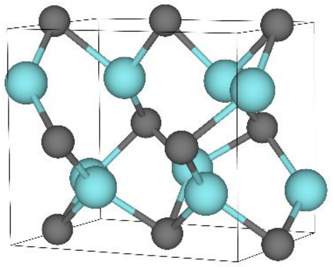

(b) $Ni_7C_4$

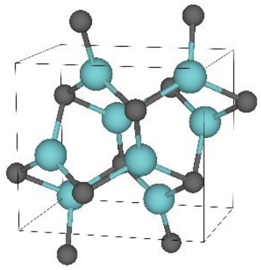

(c) $Ni_2C$ ($\boldsymbol{Pbcn}$)

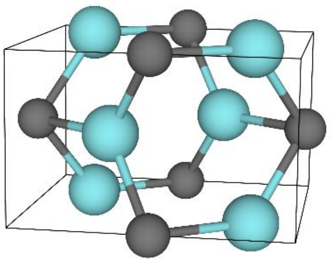

(d) $Ni_2C$ ($\boldsymbol{Pnnm}$)

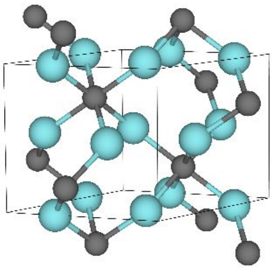

(e) $Ni_3C$ (bainite)

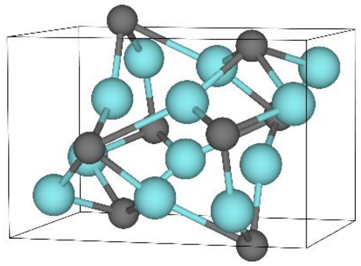

(f) $Ni_3C$ (cemenite)

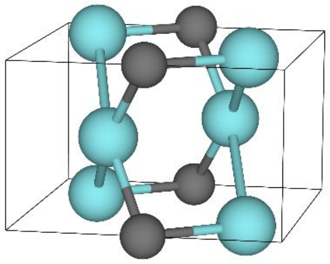

(g) $Ni_4C$

*Figure S3. Schematic illustration of the studied modified nickel $NiC_x$ lattice structures. Carbon atoms are shown in grey, and nickel atoms are depicted in cyan.*

*Table S2. Summary of the calculated zero-point energy values $E_{ZPE}$, energy contribution of the phonons $E_{Phonon}$, internal energy values U, Entropy contributions at 1073 K ($-T \cdot S$), and formation energy values $E_f$ per formula unit (f.u.) and atom (atm) for different Ni-C systems at T = 1073 K. The calculated modified lattice structures have been adopted from Gibson et al.* [1]*. All values are reported in $eV$ per formula unit, except $E_f^{atm}$, which is given in $eV$ per atom.*

| | | $E_{ZPE}$ | $E_{Phonon}$ | $U$ | $-T \cdot S$ | $E_f^{f.u.}$ | $E_f^{atm}$ |
|---|---|---|---|---|---|---|---|
| Octahedral | NiC | 0.077 | 0.407 | -8.711 | -1.135 | 1.748 | 0.874 |
| | $Ni_3C$ | 0.207 | 0.818 | -15.844 | -2.118 | 0.506 | 0.014 |
| | $Ni_4C$ | 0.228 | 1.084 | -18.576 | -2.952 | 0.651 | 0.130 |
| | $Ni_8C$ | 0.430 | 2.086 | -30.408 | -5.492 | 0.323 | 0.036 |
| | $Ni_{16}C$ | 0.770 | 3.974 | -53.291 | -10.459 | 0.451 | 0.027 |
| | $Ni_{27}C$ | 1.246 | 6.561 | -84.759 | -17.270 | 0.621 | 0.022 |
| | $Ni_{32}C$ | 1.438 | 7.760 | -99.351 | -20.480 | 0.411 | 0.012 |
| Tetrahedral | NiC | 0.057 | 0.223 | -8.764 | -0.547 | 1.834 | 0.917 |
| | $Ni_3C$ | 0.188 | 0.819 | -14.926 | -2.148 | 1.425 | 0.040 |
| | $Ni_4C$ | 0.137 | 0.926 | -17.684 | -2.669 | 1.543 | 0.309 |
| | $Ni_8C$ | 0.383 | 1.974 | -28.406 | -5.241 | 2.326 | 0.258 |
| | $Ni_{16}C$ | 0.770 | 3.974 | -51.739 | -10.468 | 2.003 | 0.118 |
| | $Ni_{27}C$ | 1.144 | 6.278 | -82.795 | -16.573 | 2.585 | 0.092 |
| | $Ni_{32}C$ | 1.372 | 7.685 | -97.840 | -20.457 | 1.921 | 0.058 |
| Modified lattice structure | $Ni_3C_2$ | 0.318 | 0.934 | -21.358 | -2.233 | 2.715 | 0.543 |
| | $Ni_7C_4$ | 0.635 | 2.441 | -48.300 | -6.204 | 2.721 | 0.247 |
| | $Ni_2C$ ($Pbcn$) | 0.189 | 0.657 | -13.110 | -1.629 | 0.365 | 0.122 |
| | $Ni_2C$ ($Pnnm$) | 0.188 | 0.658 | -13.118 | -1.641 | 0.357 | 0.119 |
| | $Ni_3C$ (bainite) | 0.211 | 0.774 | -16.098 | -1.947 | 0.252 | 0.063 |

| | | | | | | |
|---|---|---|---|---|---|---|
| $Ni_3C$ (cemenite) | 0.238 | 0.889 | -16.151 | -2.254 | 0.200 | 0.050 |
| $Ni_4C$ | 0.255 | 1.145 | -18.894 | -2.991 | 0.333 | 0.067 |

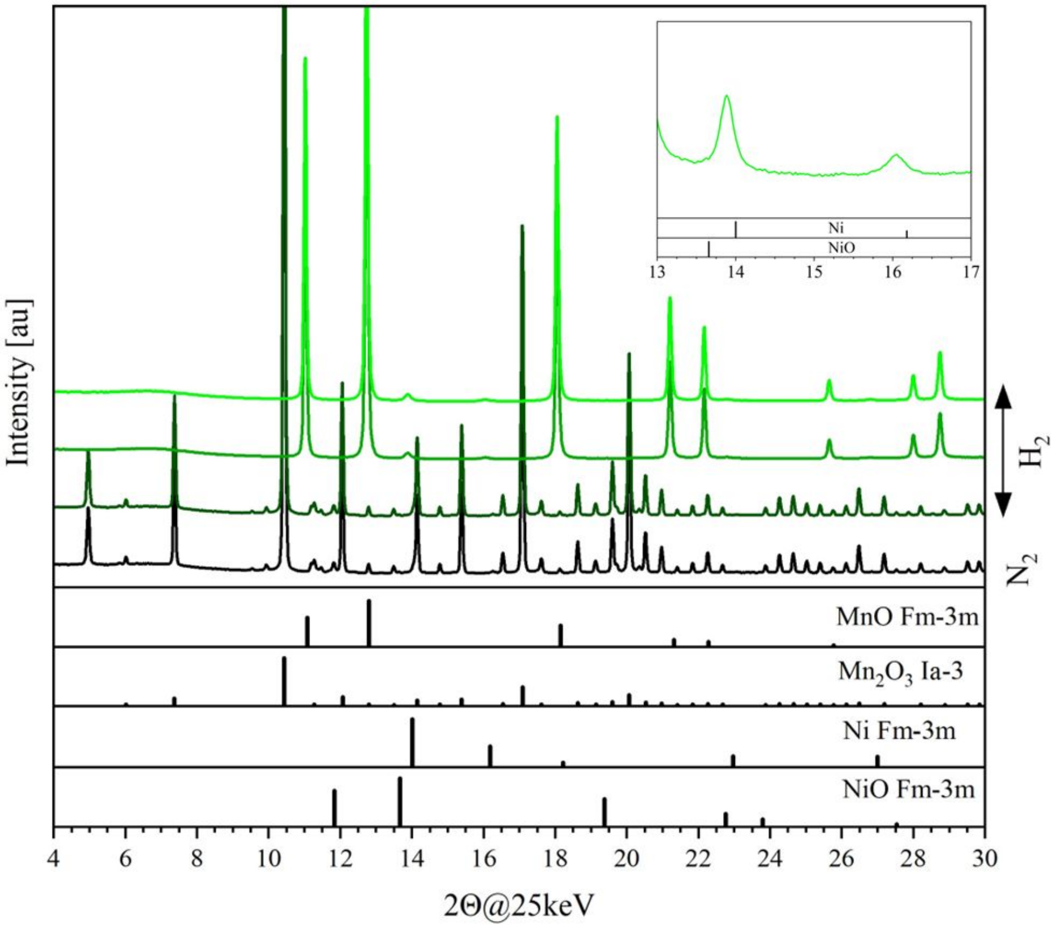


*Figure S4. The figure shows the in situ XRD patterns of the reduction step with the $NiO/Mn_2O_3$ at 500 °C and $H_2$ flow of 0.9 $NmLmin^{-1}$. The patterns (obtained at different TOS, spanning over 10 min) are stacked and progressively turn from black to green (bottom up) with time on stream (TOS). The insight panel shows a magnification of the 13-17 2Θ range of the last pattern, highlighting the absence of NiO. The reference PDFs at the bottom of the graph belong to Ni (00-004-0850), NiO (96-101-0094), MnO (01-078-0424) and Mn2O3 (96-151-4114).*

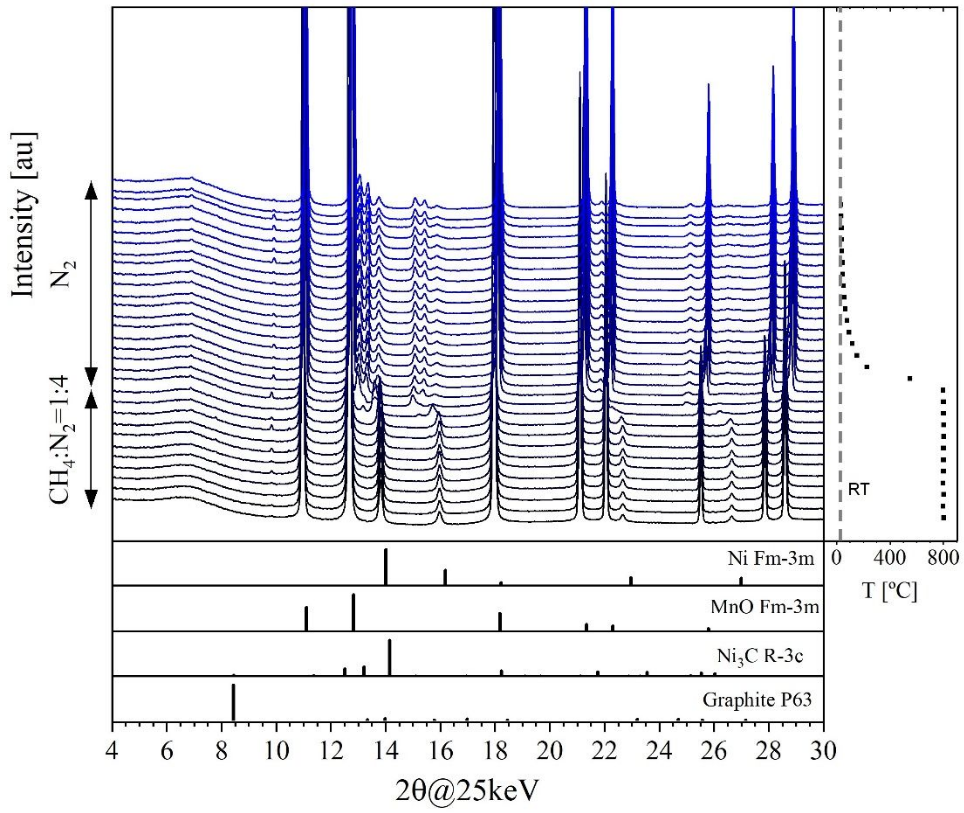


*Figure S5. The figure shows the in situ XRD patterns of the CVD experiment with the 5% Ni/MnO exposed to $CH_4$ at 800 °C, $NiC_x$ formation and later quenching to RT. The patterns (acquired with 20 s intervals) are stacked and progressively turn from black to blue (bottom up) with time on stream (TOS). The panel in the right shows the temperature of the sample, while the reference PDFs at the bottom of the graph belong to Ni (00-004-0850), MnO (01-078-0424), $Ni_3C$ (01-072-1467) and graphitic carbon (00-041-1487).*

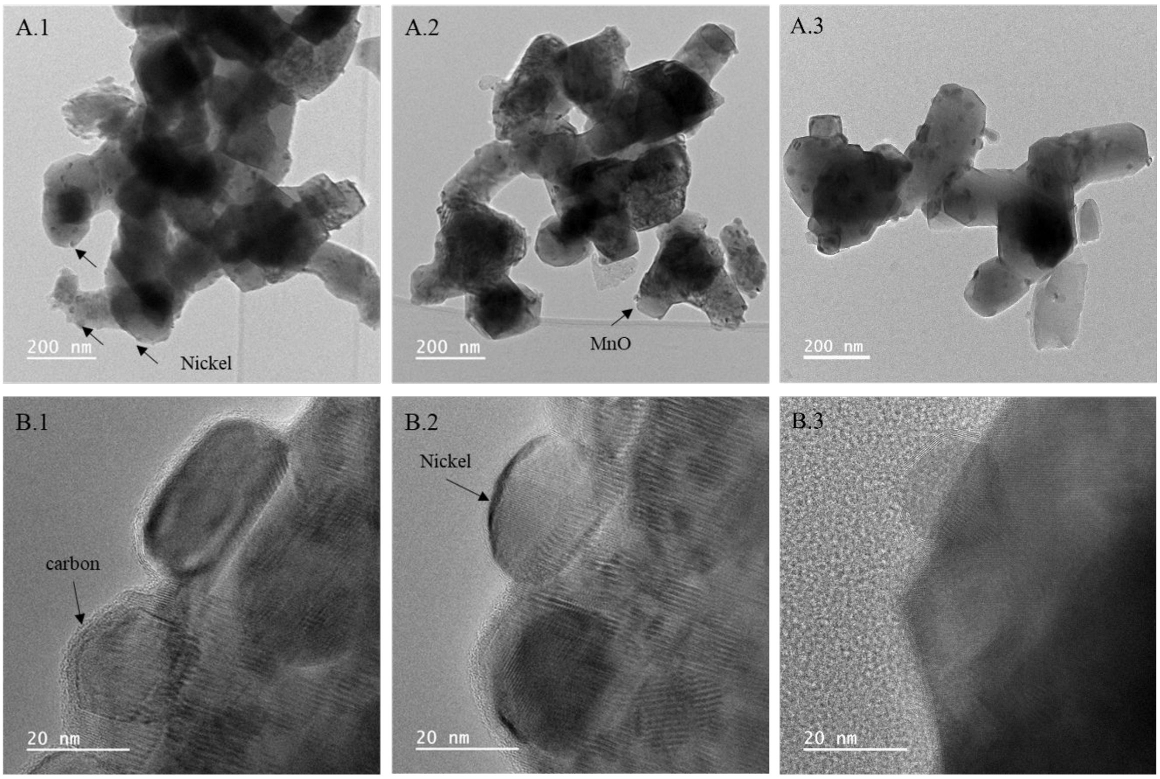


*Figure S6. TEM images of the 5% NiC$_x$/MnO material, demonstrating (A) lack of formation of carbon nanotubes/nanofibers and (B) presence of few carbon structures encapsulating the nickel nanoparticles.*

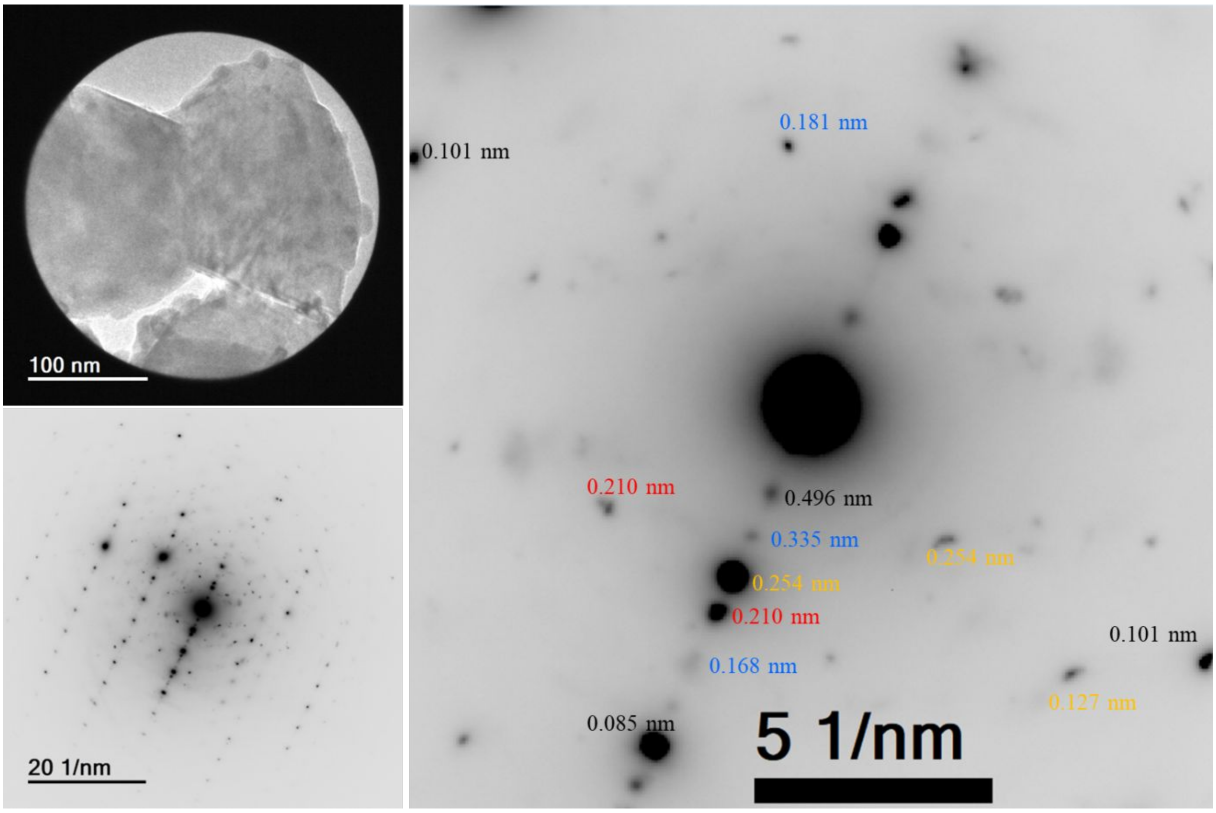


*Figure S7. SAED analysis of the $NiC_x$/MnO material. The reflections identified in red belong to $NiC_x$ (expanded d-spacings compared to $Fm\bar{3}m$ Ni, as seen in table S3), blue to graphite (0.335 nm, 0.181 nm, 0.168 nm) and orange to MnO. The black ones remain unidentified.*

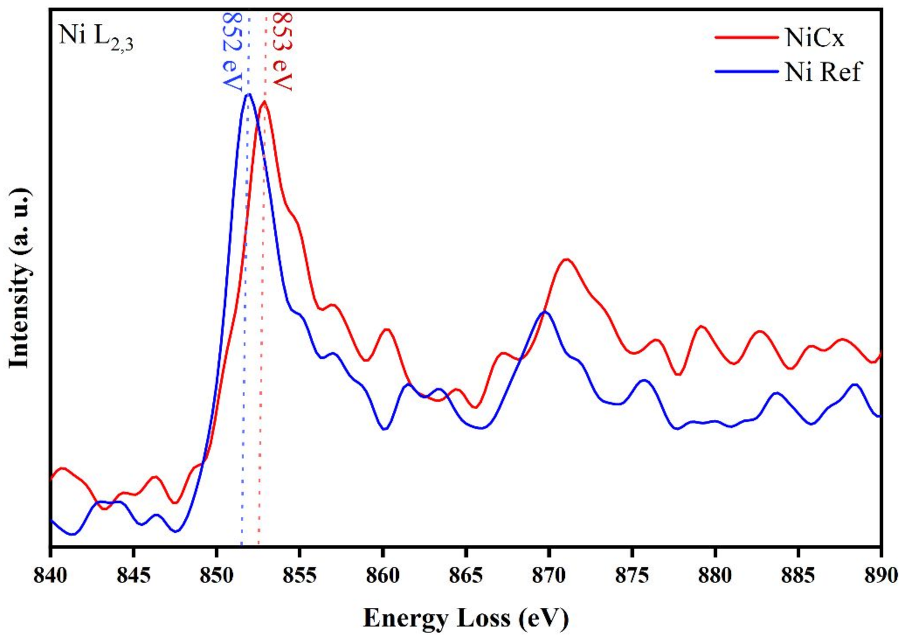


*Figure S8. Comparison of the Ni $L_{2,3}$ edge acquired on a $NiC_x$ particle and a Ni-metal reference sample. A shift of 1 eV to higher energy losses can be seen for the $NiC_x$ sample compared to the reference sample. The Ni-metal reference samples was obtained as the $NiC_x$ sample (see methods) without the CVD part, i.e. only with the reduction step leading to the 5% Ni/MnO material.*

*Table S3. Ni, C (graphite) and MnO plane distances with corresponding hkl indexes, obtained from ICDD database. Entries are: Ni (00-004-0850), MnO (01-078-0424), and graphitic carbon (00-041-1487).*

| **Material** | **d [nm]** | **(hkl)** |
|---|---|---|
| C (P63/mmc) | 0.3376 | 002 |
| MnO ($Fm\bar{3}m$) | 0.2565 | 111 |
| MnO ($Fm\bar{3}m$) | 0.2221 | 200 |
| C (P63/mmc) | 0.2139 | 100 |
| C (P63/mmc) | 0.2039 | 101 |
| Ni ($Fm\bar{3}m$) | 0.2034 | 111 |
| C (P63/mmc) | 0.1807 | 102 |
| Ni ($Fm\bar{3}m$) | 0.1762 | 200 |
| C (P63/mmc) | 0.1681 | 004 |
| MnO ($Fm\bar{3}m$) | 0.1570 | 220 |
| C (P63/mmc) | 0.1548 | 103 |
| MnO ($Fm\bar{3}m$) | 0.1339 | 311 |
| MnO ($Fm\bar{3}m$) | 0.1282 | 222 |
| Ni ($Fm\bar{3}m$) | 0.1246 | 220 |
| C (P63/mmc) | 0.1234 | 110 |
| C (P63/mmc) | 0.1160 | 112 |

| | | |
|---|---|---|
| C (P63/mmc) | 0.1121 | 006 |
| MnO ($Fm\bar{3}m$) | 0.1110 | 400 |
| Ni ($Fm\bar{3}m$) | 0.1062 | 311 |
| C (P63/mmc) | 0.1057 | 201 |
| Ni ($Fm\bar{3}m$) | 0.1017 | 222 |
| Ni ($Fm\bar{3}m$) | 0.0881 | 400 |
| Ni ($Fm\bar{3}m$) | 0.0808 | 331 |
| Ni ($Fm\bar{3}m$) | 0.0788 | 420 |

*Table S4. Comparison of experimental interplanar distances obtained by TEM imaging and XRD-RR analysis.*

| TEM | XRD-RR | | | |
|---|---|---|---|---|
| | $NiC_{0.3}$ | $NiC_{0.24}$ | $NiC_{0.05}$ | MnO |
| 1.85 Å | 1.89 Å (002) | 1.85 Å (002) | 1.79Å (002) | |
| 2.16 Å | 2.18 Å (111) | 2.13 Å (111) | 2.07 Å (111) | |
| 2.25 Å | | | | 2.22 Å (200) |

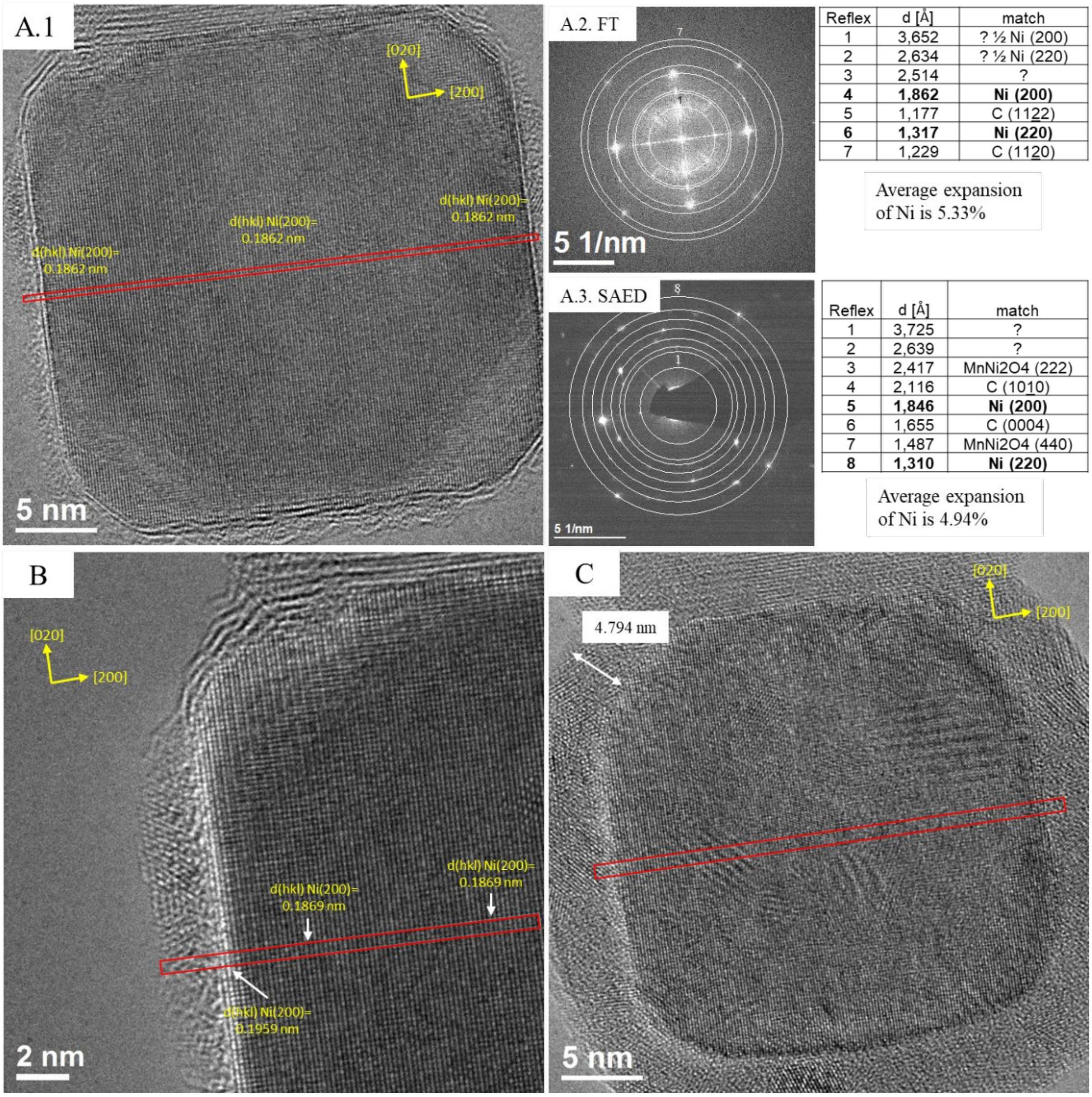


| Reflex | d [Å] | match |
|---|---|---|
| 1 | 3,652 | ? ½ Ni (200) |
| 2 | 2,634 | ? ½ Ni (220) |
| 3 | 2,514 | ? |
| **4** | **1,862** | **Ni (200)** |
| 5 | 1,177 | C (11$\underline{2}$2) |
| **6** | **1,317** | **Ni (220)** |
| 7 | 1,229 | C (11$\underline{2}$0) |

| Reflex | d [Å] | match |
|---|---|---|
| 1 | 3,725 | ? |
| 2 | 2,639 | ? |
| 3 | 2,417 | MnNi2O4 (222) |
| 4 | 2,116 | C (10$\underline{1}$0) |
| **5** | **1,846** | **Ni (200)** |
| 6 | 1,655 | C (0004) |
| 7 | 1,487 | MnNi2O4 (440) |
| **8** | **1,310** | **Ni (220)** |

*Figure S9. Stability TEM Data. A.1. A $NiC_x$ particle at the beginning of the analysis: the d-spacing belongs to an expanded Ni(200). The diameter of the particle is 33.329 nm. A.2 Fourier Transform (FT) of A.1, and A.3. small area electron diffraction (SAED) of A.1. with tables showing the d-spacings from both methods. B.1. Magnification of the nanoparticle edge from A.1, with d-spacings belonging to Ni(200). C.1. The same nanoparticle after 70 minutes of TEM-analysis. The diameter of the particle is 28.6 nm, while the external shell of ex-soluted C is 4.8 nm. The reference d-spacings of Ni can be found in Table S3. All the distances have been measured using ImageJ software[2].*

## References

(1) Gibson, J. S.; Uddin, J.; Cundari, T. R.; Bodiford, N. K.; Wilson, A. K. First-principle study of structure and stability of nickel carbides. *J. Phys.: Condens. Matter.* **2010**, *22* (44), 445503.

(2) Schneider, C. A.; Rasband, W. S.; Eliceiri, K. W. NIH Image to ImageJ:25 years of image analysis. *Nat. Methods* **2012**, *9*, 671-675. DOI: 10.1038/nmeth.2089.
